# Advancing equitable pain care worldwide requires progress in low- and middle-income settings: priorities for practice, research, and policy

**DOI:** 10.1097/j.pain.0000000000004130

**Published:** 2026-11-02

**Authors:** Saurab Sharma

**Affiliations:** a Pain Management Research Institute, Kolling Institute, Sydney Medical School, Faculty of Medicine and Health, The University of Sydney and Northern Sydney Local Health District, Sydney, NSW, Australia; b Pain Management and Research Centre, Royal North Shore Hospital, Northern Sydney Local Health District, Sydney, NSW, Australia; c School of Health Sciences, Faculty of Medicine and Health, University of New South Wales, Sydney, Australia; d Centre for Pain IMPACT, Neuroscience Research Australia, Sydney, Australia; e Department of Physiotherapy, Manipal Academy of Higher Education, Manipal University, Manipal, India

## Abstract

Equitable, high-value pain care remains out of reach for many populations worldwide, particularly for people living in low- and middle-income countries and cultural minority groups in high-income settings. Despite carrying a disproportionate burden of chronic pain, these populations remain underrepresented in high-quality pain research, underserved by existing health systems, and constrained by social, political, environmental, and commercial determinants of health. The lack of locally generated evidence forces clinicians and policymakers to rely on externally produced research that may not translate to local cultural, linguistic, or health-system contexts. This invited viewpoint synthesizes key contributors to pain inequity—including limited prioritization of chronic pain in low- and middle-income countries, underdeveloped rehabilitation capacity, poor access to essential treatments, commercial exploitation, and misalignment between culturally shaped pain beliefs and Western-derived assessment and treatment models. I highlight opportunities to strengthen equity in clinical care through improved education of health professionals and the public, culturally and linguistically responsive assessment, and contextually adapted self-management and treatment strategies. Equitable research requires standardized collection of sociodemographic variables, deliberate inclusion of underrepresented groups, and global partnerships that avoid colonizing practices. I propose principles for fair and impactful North–South collaborations focused on co-creation of research agendas, shared leadership, sustainable capacity building, and transparent governance. Advancing equitable global pain care and research will require collective action from clinicians, researchers, institutions, policymakers, journals, funders, and communities. A coordinated shift toward culturally grounded, contextually adaptable, and ethically collaborative approaches is essential to meaningfully reduce global pain disparities and ensure all individuals benefit from scientific progress.

## 1. Introduction

For many vulnerable populations in low- and middle-income settings, equitable, high-value pain care remains far from reality. The 2025 International Association for the Study of Pain Global Year defined low- and middle-income settings as populations living not only in low- and middle-income countries (LMICs) but also minority populations such as migrants, refugees, and Aboriginal populations living in high-income countries.^19^ Apart from local health systems related barriers in these settings (eg, shortage of healthcare professionals for pain care), a key contributor is the lack of context-specific pain research to inform local clinical practice and policy.^48,60,73,96,102^ Clinical care must, therefore, rely on external evidence that may not apply to the target populations, such as minority and Aboriginal peoples in high-income countries, and populations living in LMICs, or to their healthcare systems.

Between- and within-country inequity in health care and health research is also profound worldwide. In high-income countries, Aboriginal and migrant populations are often offered western ways of treatments while disregarding their traditional and Indigenous beliefs and approaches.^36^ They are also underrepresented in health research because of language, cultural, or stigma-related barriers.^73^ When included in research, their cultural, traditional, or Indigenous knowledge is often ignored.^60^ In LMICs, within and between country differences are substantial in terms of access to care, health workforce capacity, and funding in pain services and research, making each country and within country contexts unique (Box 1). For example, some upper-middle-income countries (eg, China, Brazil) and lower-middle-income countries (eg, India) have relatively greater health and medical research funding, capacity, and, therefore, more research outputs, many other LMICs must rely on external research funding, mentorship, and leadership.^92^ Despite obvious differences, many LMICs also share several common challenges and opportunities for shared solutions. This paper outlines an overarching framework and recommendations for advancing equitable pain care worldwide. Although a significant component of the paper is centred around LMIC contexts, its implications can extend to underserved populations globally, including migrant, refugee, and Aboriginal communities in high-income countries.

Box 1. **Defining low- and middle-income countries (LMICs).**

The World Bank classifies countries each year into 4 income groups based on gross national income (GNI) per capita. These thresholds are updated annually to account for inflation and global economic changes. The classification for fiscal year 2026 is as follows:

- Low-income countries: GNI per capita of US$ 1135 or less
- Lower-middle-income countries: GNI per capita of US$ 1136–4495
- Upper-middle-income countries: GNI per capita of US$ 4496–13,935
- High-income countries: GNI per capita of US $13,935 or more

The term low- and middle-income countries (LMICs) collectively refer to countries in the low-, lower-middle-, and upper-middle-income categories.

It is important to note that LMICs are highly heterogeneous, encompassing diverse geographic regions, health systems, and sociocultural contexts. Although income classification reflects economic capacity, it does not fully capture within-country inequalities, health system performance, or disease burden. In pain research, LMICs often face disproportionate challenges, including limited access to pain management services, workforce shortages, and constrained health resources.

Note: for up-to-date classification of LMICs, please refer to the World Bank website (https://blogs.worldbank.org/en/opendata/understanding-country-income--world-bank-group-income-classifica).

## 2. Reflexivity statement

Reflexive awareness of the author's worldview is essential for appropriately contextualizing the paper's framing, recommendations, and conclusions. This author identifies as male, was born and raised in Nepal, and has nearly a decade of experience there as a clinician and educator. He completed his Bachelor's and Master's degrees in Physiotherapy in India over 6.5 years. The author then completed a PhD from the University of Otago in New Zealand with most work focusing on pain research in Nepal. He has personal experience of chronic headache throughout most of his life, along with recurrent musculoskeletal pain, including low back pain, over the past 2 decades. He is a full-time researcher and has been based in Australia for the past 4.5 years. His research primarily focuses on improving equity in pain care and research for vulnerable populations, particularly those living in low- and middle-income settings. His lived experience of pain, clinical practice, and experiences of living in 2 LMICs and as an ethnic minority person in 2 high-income countries have shaped his research trajectory and have both directly and indirectly informed this manuscript and the views expressed.

## 3. Defining equitable care and research

*Equitable care* is a widely used but inconsistently defined term. For this review and beyond, equitable care is defined as the provision of fair and just opportunities to receive care or participate in research, accounting for individuals' unique circumstances and needs.^99,103,109^ In pain care, achieving equity requires consideration of peoples' clinical, social, contextual, cultural, and linguistic backgrounds, which shape pain experiences and consequently outcomes.^59,82,90,109^

Equitable care goes beyond “equal” care. It prioritizes meaningful improvements in health outcomes by identifying and removing barriers to accessing effective, safe, and affordable care, and intentionally addressing the social and systemic inequities that shape pain experiences and care pathways. *Equality*, by contrast, implies offering identical services to all individuals regardless of need—an approach that risks perpetuating disparities when the individual's context is ignored.

## 4. Contributors to inequitable pain care and research

### 4.1. Chronic pain is not a priority in low- and middle-income countries

Chronic pain remains underprioritized in health care and research in LMICs. Low- and middle-income countries face competing priorities and policy agendas, such as communicable diseases, maternal and child health, and the growing burden of noncommunicable diseases, which dominate attention and resource use.^18,83^ Although Global Burden of Disease studies consistently rank chronic pain conditions, particularly low back pain, as a leading cause of disability, primary country-level data remain limited in many LMICs, hindering meaningful local policy responses.^102^

Chronic pain prevalence in LMICs is often comparable to, or higher than, that in high-income countries.^41,46,47^ Underreporting is common in settings where chronic pain is normalized as a feature of ageing or everyday life, or is interpreted through cultural, spiritual, or religious beliefs such as pain as a consequence of actions in the past life, contributing to unhelpful acceptance and avoidable suffering.^87^ In addition, heterogeneity in survey methods and pain measurement instruments across studies produces variable prevalence estimates and complicates cross-country comparisons. Internationally applicable, culturally acceptable, and standardized chronic pain epidemiology questions are needed to improve data quality and support meaningful and fair comparisons across settings.

### 4.2. Disproportionate global pain burden and inequitable access to effective care

Pain burden is unevenly distributed across populations and is most pronounced in low socioeconomic contexts (eg, LMICs and migrant or Aboriginal peoples in high-income countries).^37,46,96^ Despite global progress in pain research, education, and advocacy, only a small proportion of high-quality pain research originates from LMICs, as well as from migrant and Aboriginal populations in high-income countries. Chronic pain remains a leading contributor to years lived with disability worldwide, and a disproportionate share of this burden is attributed to LMICs, driven largely by population growth and aging.^37–39^ Low- and middle-income countries are, therefore, likely to carry the greatest disability load in the coming decades. Similarly, Indigenous peoples, refugees, and culturally diverse migrant populations in high-income countries hold pain-related beliefs that differ from those of dominant cultural groups, often reflecting their Indigenous or cultural identities and community values.^36^ These beliefs remain unaddressed by western approaches to pain management. As a result, these populations consistently experience a greater burden of pain and are more likely to receive poor care.^12,28^

Timely, coordinated action is needed to halt or reverse the growth of pain-related global disability, with targeted support for LMICs and marginalized populations in high-income countries. People living in LMICs experience structural constraints such as inadequate infrastructure and resources and limited access to essential treatments and rehabilitation services that impede effective care.^16,17,88^ Although the burden and management of pain vary within and between countries, an array of shared drivers underpin pain-related inequity.^95,107^

### 4.3. Contributions of social, political, environmental, and commercial determinants

Inequities in pain research and care in low- and middle-income settings arise from complex interactions of social, political, environmental, and commercial determinants. These factors shape who develops chronic pain, who receives guideline-concordant assessment and treatment, and who is represented in the evidence base.^40,107^ These contemporary determinants are deeply rooted in historical and structural forces, including colonization, systemic racism, and their enduring effects on political stability, health systems, and research capacity in many countries (both high-income countries and LMICs).^36^

In some contexts, health systems and research institutions may be perceived as extensions of colonial or externally imposed models, further influencing trust, engagement, and the perceived relevance of care and research. Collectively, these determinants can widen the inequity gap and compromise the quality of pain assessment and treatment, especially for vulnerable and marginalized populations such as the migrants, cultural minority groups, refugees, and Aboriginal peoples.^20,95,107^ Scientifically, these same factors also influence research agendas—what is funded, who is included (or excluded), and how results are produced and reported—ultimately reinforcing inequities in evidence base.

People's social positioning influences the entire clinical pathway, from recognition and communication of pain through referral, treatment, and outcomes. Persistent disparities across race/ethnicity, sex, age, socioeconomic position, and culture indicate that inequities are not confined to a select few health systems, but instead reflect entrenched social and political determinants worldwide.

At the political and health systems level, the availability of essential analgesics remains limited for acute pain and palliative care, while rehabilitation capacity is constrained. For example, the World Health Organization reports 5- to 63-fold differences in medical morphine consumption between countries, illustrating a persistent mismatch between population sizes, need, and access.^105^ Similarly, built environments in many LMICs lack safe public spaces, walkability, or transport, making it harder to put into practice recommended pain treatment such as physical activity and outdoor exercise, which are not just affordable but also easily implemented.^18^

In contrast, commercial influences can promote the use of costly and potentially harmful investigations and treatments, often marketed as innovative despite lacking robust evidence. Although no countries or settings are immune to commercial influence in health and research, LMICs are particularly vulnerable.^40^ The population size of LMICs, ie, ∼85% of the world's population, is considered as business *markets* with great potential for growth. Marketing strategies include training healthcare professionals in “novel” treatment approaches, convincing them that these are effective approaches, often using small or poor-quality studies. These therapies often promise quick fixes and are offered before exhausting guideline-recommended treatments such as education, exercise, or psychological therapies. In the context of many LMICs, pain treatments are out-of-pocket costs, putting tremendous financial strain on individuals and societies, further widening inequity. Figure 1 schematically summarizes the framework for addressing these determinants.

**Figure 1.**
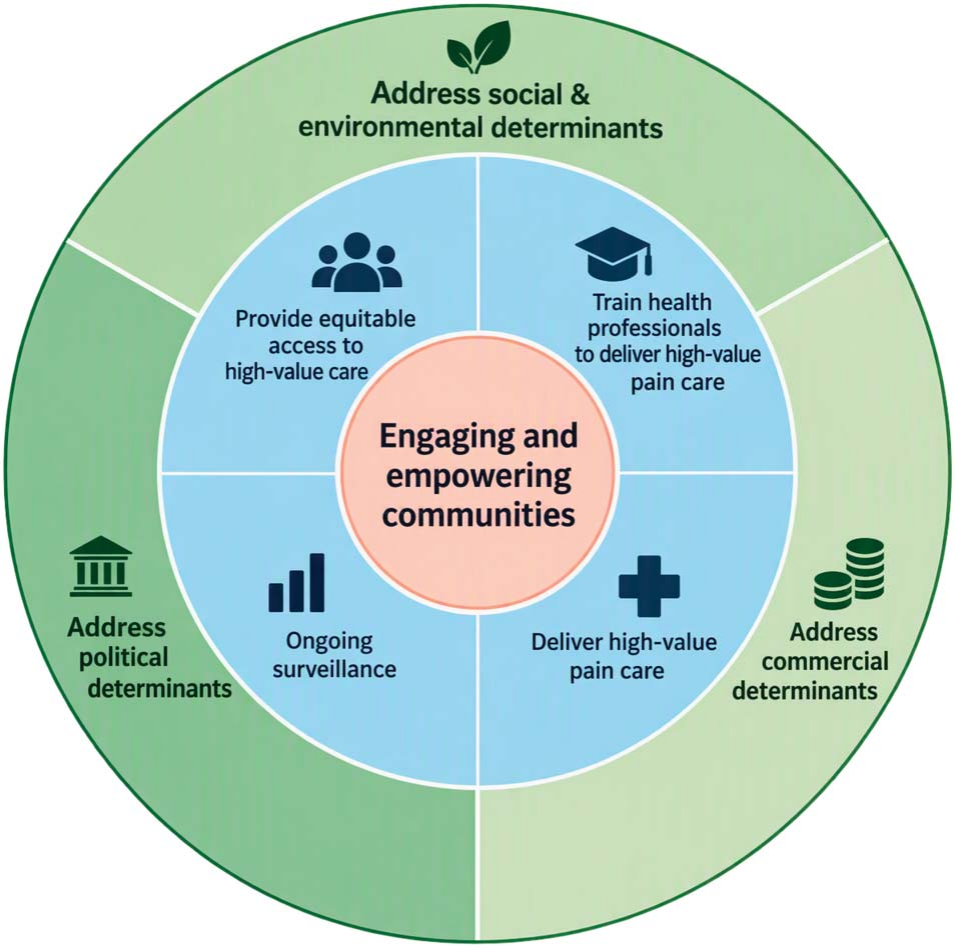
Framework for addressing social, political, environmental, and commercial determinants of pain.

## 5. Towards equitable pain care across populations

Achieving equitable pain care requires a multifaceted approach, ranging from improved education for healthcare professionals and the public to clinical and policy strategies for pain assessment and treatment. It is important to practice strength-based approaches where applicable, eg, to embrace traditional and spiritual approaches to pain care that have been practiced in specific target settings, subjecting them to rigorous research where warranted and feasible, and disseminating such approaches for international testing and uptake. Importing treatment principles or approaches from other settings may be needed in some contexts. Strategies for advancing equitable pain care with exemplary actions are presented below (Fig. 2).

### 5.1. Educating healthcare professionals and public in pain

Healthcare professionals' education on pain is currently suboptimal in their training curricula. The gaps in training can vary largely between countries from lack of training around cultural sensitivity in pain care (eg, high-income countries) to complete avoidance of biopsychosocial framework of pain (eg, in some LMICs). Healthcare professionals' education must adopt or adapt standard recommended curricula, eg, the one recommended by the International Association for the Study of Pain.^44^

Healthcare professionals' education must include training in person-centred, culturally responsive, and trauma-informed care with shared decision making, to serve diverse populations and meet individual patient needs. There is evidence from Brazil to show that training of healthcare professionals in pain management using written or video-based resources improves overall pain knowledge and confidence.^2,64^ Healthcare professionals require upskilling to deliver effective pain care within local contexts. Locally relevant, open-access educational resources are emerging to support both clinicians and people with lived pain experience.^76^

Various avenues remain for upskilling healthcare professionals. A good example of a resource for upskilling healthcare professionals is the Essential Pain Management^65^ courses that has been delivered in more than 60 countries worldwide, and now available online (https://praxhub.com/organisations/essential-pain-management-53). The more recently introduced Online Pain Education Network (OPEN) Pain Training Programme (https://openpaintraining.com) uses asynchronous approach to education and has the capability for the greatest reach, reducing access barriers, and upskilling healthcare professionals and students at scale. One barrier, however, for those with limited resources, is the costs associated with its access.

Similarly, public education to support evidence-based pain self-management is necessary. In LMICs, a promising approach may be to target school children, who can act as effective conduits of health information to their families. Proof-of-concept studies from Zambia^13^ and Kenya^72^ in the context of handwashing interventions have demonstrated that health education provided to children can generate spill-over effects, influencing the health behaviors of their family members and broader communities. These benefits may also be sustained over time, suggesting the potential long-term population-level impacts.^11,56^ Although such studies are currently unavailable in the pain field, this approach may be worth testing and could be particularly useful in contexts where parental or community educational levels are low. This may apply to LMICs as well as socioeconomically disadvantaged migrant, refugee, and Aboriginal communities in high-income countries. In addition, publicly available pain educational resources on credible websites (such as national government websites and international organization's websites) are needed in multiple languages, to educate patients and public at scale.^7^

### 5.2. Equitable assessment of people with pain

People's cultural backgrounds, sociodemographic and economic contexts, and upbringing shape their beliefs about health and pain, as well as how they communicate pain, including their descriptions of pain quality and severity.^33,34,77,82,85,90,98,101^ These beliefs in turn can impact how people cope with their pain such as by ignoring it, worrying, resting or pushing through it, or by seeking help or isolating.^90^ Pain beliefs also influence treatments people seek and receive, ultimately influencing treatment outcomes. Therefore, understanding patients' unique pain beliefs through their historical and cultural lens is critical to address in pain treatments.^36^ Not by dismissing them, rather by acknowledging their beliefs, and guiding them toward evidence-based approaches to pain self-management and treatment.^82,87^

The language of pain is also important to consider. For example, people with chronic pain in some South Asian countries like India and Nepal use unique descriptors to describe their pain that is not commonly used or comprehended by individuals from western cultures (eg, the United States).^8,85,98^ They describe pain using distinctive embodied expressions—eg, *jham-jham* (or *jhum-jhum*) or *kat-kat*—that convey qualitative sensations related to pain. These terms are analogous to *onomatopoeic* expressions to describe sounds in English (eg, *tik-tok* for the sound of clock ticking). Such distinct expressions of pain in some South Asian cultures are culturally and historically embedded and generationally transmitted. These pain expressions are also not easily translatable into English because they encapsulate culturally specific ways of perceiving and communicating bodily sensations and experiences. As such, these pain expressions are rare in western contexts and have been misinterpreted as neuropsychiatric disorders.^55^ These words often describe specific sensations, for example, *jham-jham* (or *jhum-jhum*) is used in India and Nepal to describe tingling or paraesthesia.^8,68^ Similarly, the term k*at-kat* is used in Nepal and North India, but with nuanced differences in its meaning (Nepali individuals describe *kat-kat* as an achingor cold sensation, whereas North Indian populations use *kat-kat* to describe crepitus).

The language people use to describe their pain matters. People with pain have a fundamental need to feel heard and understood, and manyswitch healthcare providers when this needgoes unmet. Indeed, some individuals travel internationally not only to access better care, but also to find healthcare professionals who can interpret the cultural meanings embedded in their pain descriptions. For example, a Nepali adult with knee pain was reported to have travelled from the United Kingdom back to Nepal to consult healthcare professionals who understood their experience of “*kat-kat*” pain.^108^ This highlights the importance of timely referral to healthcare professionals who understand patients' linguistic and cultural pain expressions, or alternatively, the need for healthcare providers to actively seek this understanding in routine care. Consequently, culturally relevant patient-reported measures must be co-developed from the ground up with patients experiencing pain and healthcare professionals—particularly for pain constructs that are culturally rooted or heavily influenced by cultural interpretation—rather than relying solely on translated English-language tools that may lack content validity.^85,98^

Deliberate selection of appropriate measures to assess pain and psychosocial domains is also needed for equitable assessment of people with pain. Commonly used pain assessment tools—such as the Numerical Rating Scale (NRS) and Visual Analog Scale (VAS)—may not be suitable across all cultures and literacy levels. Cumulative evidence from at least 3 Asian countries (Bhutan, China, and Nepal) shows that age and education significantly influence the usability and acceptability of these scales.^58,77,78,101^ Although all standard measures (NRS, VAS, Verbal Rating Scale [VRS], and Faces Pain Scale–Revised [FPS-R]) demonstrate adequate construct validity, NRS and VAS produce higher rates of incorrect responses, particularly among older adults and those with fewer years of education. In contrast, FPS-R remain the most preferred and acceptable scale among multiple cultural groups including Bhutanese, Brazilian, Hmong, Nepali, Kenyan, and Sri Lankan individuals,^32,42,61,62,77,80,101^ followed by the VRS (or its pictorial vertical modification to represent a thermometer among Chinese individuals).^58^ These scales, therefore, have greater utility especially in older adults and individuals with lower education attainment, as these are also associated with fewer response errors in these populations.^77,101^

Attempts have been made to codesign culture-specific pain intensity scales. For example, a codesign with Hmong people with low English proficiency used a six-face format similar to FPS-R, but incorporated culturally meaningful colors to convey pain (and illness).^62^ However, its measurement properties have not yet been evaluated, and its applicability to other cultural groups remains unexplored.

Despite growing evidence for preference of non-numerical pain intensity scales, clinical trials and healthcare professionals continue to use the NRS. A survey of Brazilian physiotherapists revealed a mismatch between clinician preference and patient usability. Only 60% of physiotherapists could differentiate among the 4 common pain scales, and just over half of those identified them correctly.^80^ These findings highlight that even healthcare professionals may lack familiarity with scale formats, reinforcing the need for healthcare professional education and careful selection of outcome measures for the target populations consider their age, cognitive ability, and educational levels.

Together, these findings challenge the routine, uncritical use of NRS and the VAS as default measures and call for reassessment of their recommendations by the core outcome sets.^24,30^ Scale anchors such as “worst imaginable pain” or “worst pain possible” may also lack cultural relevance in some settings.^86^ Response burden should additionally be considered—5 response options are generally less cognitively demanding than 7, making them more suitable for older adults and individuals with limited education.^78^ Furthermore, assessing “average pain” can be challenging when recall periods are long or symptoms fluctuate, and this should also be accounted for in both clinical and research contexts.^25,74^ For example, in a South African study using Brief Pain Inventory (which uses numerical rating scales) reported mean scores of 3.9/10 for “average pain” and 4.6/10 for “least or minimum pain.”^74^ Conceptually, minimum pain intensity would be expected to fall below average pain, questioning the understanding and validity of participants' responses on the scale. Equitable pain assessment, therefore, requires tools that are culturally and contextually appropriate. Faces Pain Scale–Revised or Verbal Rating Scale may be more suitable first-line options in diverse or lower-literacy settings.^32,58,61,62,77,80,101^ For research or clinical use over telephone, VRS is preferable, given that FPS-R cannot be administered over a telephone call. The use of VAS should be stopped.

### 5.3. Equitable pain treatments

Proponents of evidence-based practice rightly argue that pain care should rely on the best available evidence. However, in many LMICs, the key challenge is the limited availability of locally generated evidence and difficulty of translating external evidence into local practice. Consequently, healthcare professionals often rely on research developed from high-income Western countries, which may not align with the linguistic, cultural, social, and health system contexts of LMICs. Differences in healthcare infrastructure, language, pain beliefs, and coping strategies further constrain the meaningful application of such evidence to local populations.

A recent study of 32 LMICs showed an excessive reliance on pharmacological treatments and biomedically oriented treatments such as electrotherapies, manual therapies, and procedural interventions for (low back) pain.^95^ Social connections are important positive factors for people with pain in LMICs. In terms of self-management, people living with pain in LMICs frequently use passive self-management strategies, such as resting, herbal massage, or over-the-counter medications, whereas the use of active self-management approaches appear uncommon. Unsurprisingly, healthcare professionals also rarely integrate structured self-management support into clinical practice.^23^ In some settings, the very concept of clinician-supported pain self-management is new, as demonstrated in Ethiopia, where healthcare professionals reported limited awareness of self-management principles or how to foster patients' confidence in managing chronic pain.^23^ By harnessing social connections and empowering patients with self-management skills, interventions may support adaptive pain beliefs, promote effective coping, and better align evidence-based advice with local contexts. Further research is needed to identify the key composition of effective self-management plan and to establish how these can be embedded in pain care in LMICs.

Pain care in many LMICs relies heavily on traditional medicine and new therapies shaped by commercial interests. As a result, current practices often diverge from best-practice recommendations.^95^ Encouragingly, the number of studies evaluating feasibility and adaptation of western pain management approaches in LMICs have increased in recent years, with emerging evidence of promise.^4,5,43,84,89^ Identifying which components are culturally and contextually relevant will enable future studies to prioritize the most effective elements, thereby informing the development of treatment models suited to local needs. Early evidence also suggests that adapted interventions may improve health outcomes, as observed in musculoskeletal pain programs in Brazil.^2^

Optimal pain care in low- and middle-income settings may require integrating evidence-based Western approaches with traditional medicine that are safe, culturally meaningful, and acceptable. However, some traditional practices (eg, blood-letting) may be ineffective or harmful and should be subject to careful regulation pending high quality on their benefits and risks.^95^ When local guidelines are unavailable or not evidence based, international guidelines such as the low back pain guidelines by the World Health Organization^104^ can be useful to inform treatment decisions. However, the generation of robust local and culturally relevant evidence remains essential, as their content and delivery require careful contextual adaptation. For example, the advice to “remain active” or early return to work is central to low back pain care; however, in settings where manual labor or physically demanding work contributes to ongoing pain, such guidance may be counterproductive. How this advice should be modified—for instance, whether to encourage “relative activity” or short periods of “relative rest”—has not been adequately explored.

#### 5.3.1. Models of pain care delivery

Predominant specialist-led pathways continue to impede high-value pain care in many LMICs. A mixed-methods study across 32 countries found that orthopedic surgeons, neurologists, neurosurgeons, rheumatologists, and pain physicians frequently serve as first-contact providers for low back pain.^95^ This pattern reflects entrenched professional hierarchies, shortages of allied health professionals, and limited trust in nonphysician providers by physicians and communities. As a result, patients are often directed toward early and unnecessary imaging, procedural interventions, and surgical treatments.^4^ Chronic back pain is typically managed in tertiary care settings, further straining overstretched healthcare professionals and already burdened health systems. Strengthening the capacity of allied health professionals and primary care practitioners through training in evidence-based pain care, alongside empowering them to function as first-contact practitioners, is needed in LMICs. In settings where allied health professionals are scarce, traditional medicine practitioners and community health workers, who already serve as primary care providers in many LMICs,^95^ could be trained to support the delivery of evidence-based primary care for chronic pain.

Interdisciplinary care is recommended for managing chronic, complex pain,^22,67^ yet many LMIC health systems lack the resources and infrastructure needed to implement it effectively. This quote from previously cited mixed methods study underscores the extent of unmet needs in pain care in some LMICs, “*They [patients] go to one of the neighboring countries… [for low back pain care], if they can afford [it]*.”^95^ Although many LMICs suffer these constraints, interdisciplinary care models have been adopted in some LMICs when appropriate and awareness of the need for this approach exists. For example, Malaysia has a long-standing successful model for delivering multi/interdisciplinary care.^21^ Evidence from effectiveness and cost-effectiveness studies can help identify trade-offs and guide health system strengthening efforts in LMICs. A viable care model is community-based and lay health workers–led approach, which has shown some promise across several LMICs for a range of health conditions.^57,96^ This model may also be relevant for Aboriginal, Indigenous, and migrant minority populations in high-income countries, which needs testing.

Digital health is a promising model of care for LMICs and rural/remote areas in high-income countries. However, its implications for equitable pain care are double-edged: widespread access to digital and social media platforms enables dissemination of evidence-informed education, yet misinformation often spreads faster and extensively than credible information.

The growing influence of social media “influencers” and increasing reliance on artificial intelligence for health information warrant rigorous scrutiny, akin to evaluation applied to new members of the clinical workforce. To safeguard the public, government health websites could endorse, or blacklist digital pain-related resources based on information quality, although credible resources on pain for the public are currently lacking for several LMICs.^7^ At the same time, reliance on digital health alone risks exacerbating inequities, as people without reliable devices and internet, or adequate e-health literacy may be excluded from both pain care and research participation. Ensuring equitable access, therefore, requires blended models that combine digital strategies with accessible in-person and offline options to avoid widening gaps in care and participation (Figure 2).

**Figure 2.**
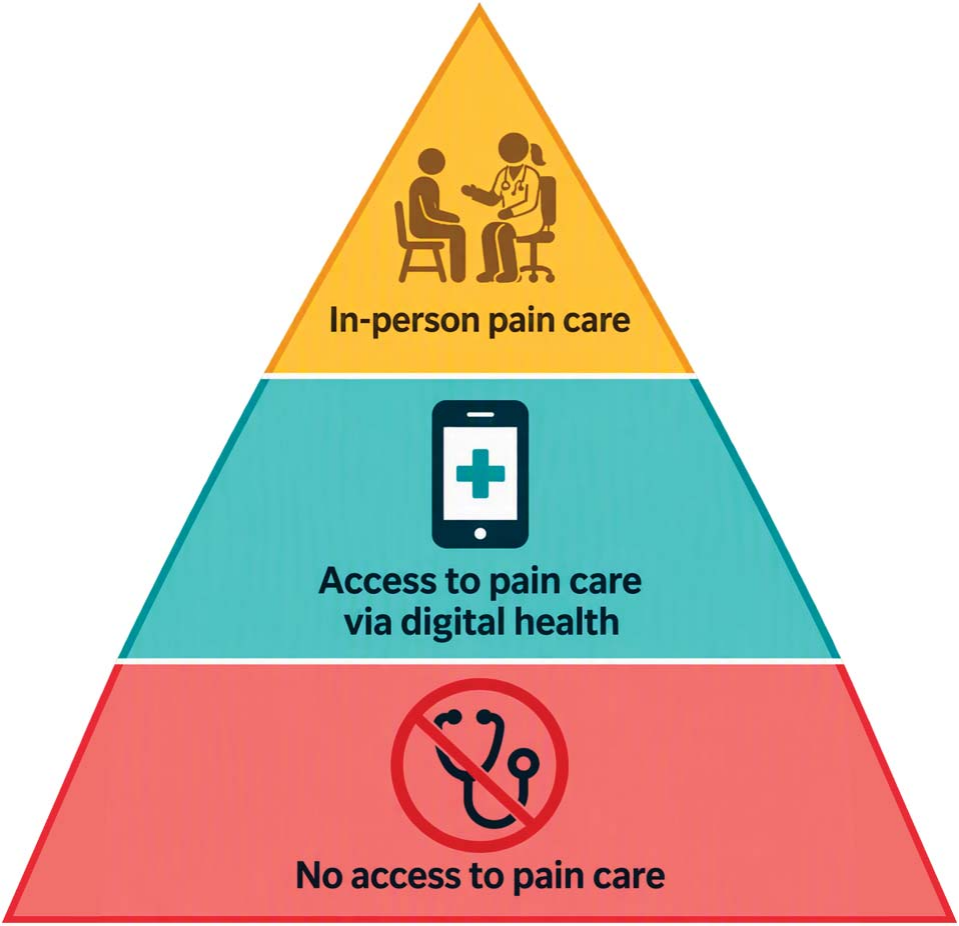
Schematic for Access to pain care in low- and middle-income settings. Note that in-person care at the top of the pyramid does not represent better care. Rather it indicates that fewer people can have access to in-person care compared to care via digital health; and many more people do not have access to any pain care.

## 6. Towards equitable pain research

Achieving equity in pain research requires identifying and addressing research-related barriers through education, capacity building, and systematic collection of equity-relevant data. It also involves meaningful inclusion of minority, diverse, and underrepresented populations, learning from other fields to adopt effective treatment models while avoiding known pitfalls, and supporting high-quality research. Strategies to achieving equitable pain research are discussed below.

### 6.1. Identifying, acknowledging, and addressing barriers to pain research

Low- and middle-income countries and minority populations living in high-income countries are underrepresented in pain research.^48^ Despite having a long history of pain research in many high-income countries, research agenda continue to focus on people from dominant backgrounds (eg, those from western or Caucasian backgrounds) and rarely address the needs of Aboriginal peoples, migrants, and other minority groups. This limitation of current pain research needs acknowledging and addressing (see Sections 6.3 and 6.4 and Table 1.)

**Table 1:** Problems, barriers, actions, and solutions for each interest holder group for improving equitable pain care and research.

Problem or barrier	Action or solution	Interest holder
Inequitable care and evidence: social position (race/ethnicity, culture, sex, age, SES, migration, Indigeneity) shapes recognition, referral, treatment, and outcomes; structural constraints (essential medicines, rehab capacity, built environment) reinforce disparities	Mandate equity targets in service access (eg, wait-time equity, language access), finance essential analgesics, and basic rehab capacity; invest in safe public spaces/transport to enable activity-based care	Policymakers and health systems
	Integrate culturally responsive, trauma-informed, person-centered care; implement interpreters, culturally adapted education, and navigation pathways	Healthcare professionals, health services
	Embed equity analyses, stratify outcomes by social factors, and codesign with communities to ensure contextual fit	Researchers
Mismatch of western evidence with LMICs and minority contexts in high-income countries: local evidence is scarce; imported guidelines and interventions often misfit linguistic, cultural, workforce, and economic realities (eg, “remain active” in manual-labor contexts)	Adapt and document intervention components; test “relative activity/rest,” delivery modes, and cost-constrained models; publish adaptation frameworks	Researchers and clinicians
	Produce context-adaptable guidance; provide companion toolkits for low-resource settings (primary/community care)	Guideline bodies; professional societies national, regional, and international pain organizations
	Prioritize pragmatic, culturally adapted trials and implementation research in LMICs and in underserved groups in high-income countries	Funders
Self-reported measures of pain and other domains important to assess in people with pain may not be fit-for-purpose: common pain and disability scales can be error-prone—especially in older and low-literacy groups	Prefer Faces Rating Scale-R or Verbal Rating Scale in diverse/low-literacy settings or in older adults Use Verbal Rating Scales for phone follow-up Shorten recall periods where feasible Reduce response options to lessen cognitive burden (ideally no more than 5 responses)	Healthcare professionals and researchers
	Codesign culturally valid patient-reported outcome measures (beyond translation) with equal partnerships with interest holders with lived experience of that culture	Researchers
	Train clinicians on measure selection/administration and interpretation across cultures	Educators
Underuse and poor integration of best evidence into practice	Systematically embed self-management support (goal-setting, pacing, problem-solving), with culturally tailored materials	Healthcare professionals and health services
	Include evidence-based treatment approaches including education and self-management in curricula and continuing education courses Upskill and assess capability in routine care	Educators and professional bodies
	Determine feasibility, acceptability, scalability of self-management models suitable for low-resource settings (eg, group/community delivery, digital health)	
Commercial determinants driving low value care: aggressive marketing and private incentives promote unnecessary imaging, procedures, and “quick-fix” therapies; out-of-pocket costs exacerbate inequity	Regulate advertising/claims Require value and safety disclosures Blacklist/endorse online resources via quality criteria posting trustworthy information on credible website government, institutional, or organizational websites	Policymakers/regulators
	Deimplement low-value care and fund high-value first-line options	Payers and health systems
	Use shared decision making; disclose uncertainty and costs	Healthcare professionals
Digital divide: digital channels can scale education/recruitment but risk excluding of those without devices/bandwidth/e-health literacy; misinformation spreads faster than evidence	Offer online + offline alternatives. Zero-rating/low-bandwidth options Monitor equity of uptake	Health systems, researchers
	Curate and endorse high-quality multilingual pain resources Counter misinformation Invest in digital inclusion	Government and public agencies
Inequitable research participation: “Equal access” approaches exclude nondominant language speakers, people distant from sites, older adults, and those with multimorbidity; clinic-based recruitment and language-only eligibility drive selection bias	Use multilingual materials (including AI-assisted), community partnerships, mobile/remote recruitment, flexible visit windows, lay facilitators, and navigators; budget for interpreters/translation	Researchers
	Encourage and reward language-based inclusions Enforce equity-related statement in funding calls	Funders and ethics boards
	Enforce reporting of equity-relevant information (just like recent uptakes around public and patient involvement statements)	Journals
Missing equity-relevant sociodemographic data	Implement the consensus minimum/optional equity datasets; prespecify subgroup and moderation analyses	Researchers
	Make standardized equity data collection/reporting a condition of publication/funding	Journals and funders
“Colonizing” research practices and tokenistic authorship: LMIC partners under-represented as authors in work done in their settings; power, budgets, decisions concentrated in HIC institutions; limited local leadership roles	Establish cogovernance (joint PIs), shared budgets, coleadership, shared expertise Invest in gaining mutual trust Clear authorship plans placing LMIC investigators in lead roles commensurate with contribution	Researchers and research networks/consortia
	Require transparency statements on governance, budget allocation, authorship distribution, and community compensation Discourage and if needed desk-reject parasitic authorship	Journals
	Replace blanket data export restrictions with fair data-sharing frameworks that protect sovereignty and enable collaboration	LMIC governments
	Reward ethical collaborations and leaderships (eg, recognizing middle authorships to facilitate LMIC capacity development)	Funders and institutions in high-resourced settings

LMICs, low- and middle-income countries; SES, socioeconomic positioning.

Low- and middle-income countries face multiple interrelated barriers to pain research.^27,75,91–94,100,106^ At a structural level, awareness of the importance of research is low among healthcare professionals, academics, policymakers, and the public. Consequently, research—and pain research in particular—is frequently underprioritized, underfunded, and inadequately supported at institutional and national levels.^92^ Many academic institutions lack a strong research culture, resulting in limited infrastructure, protected research time, mentorship, incentives, and opportunities for rigorous research. Research funding is often scarce, and when available, little is allocated to pain research. Together, these factors contribute to a limited research workforce, reduced research capacity, low or poor-quality research output across many LMICs.

Foundational infrastructure for clinical pain research is also often lacking. Many LMICs do not have access to reliable, valid, and responsive patient-reported outcome measures that are culturally adapted and available in local languages. This limits the feasibility and quality of epidemiological and clinical pain studies.

Capacity constraints further compound these systemic challenges. Many healthcare professionals and academics have limited training in research methodology, study design, and statistical analysis. Scientific writing in English pose additional challenges for many non-native speakers, particularly in conveying subtle but important distinctions in terminology such as language implying association vs causation.^3^ Collectively, these limitations can compromise research quality and reduce the untrustworthiness of research findings.

Despite limited funding and institutional support, academics in LMICs are expected to publish for career advancement. When pressure to publish is combined with inadequate research training and mentorship, researchers in LMICs become more vulnerable to predatory publishing practices compared with their counterparts in high-income countries.^92^

Addressing these challenges requires coordinated, multilevel strategies. These include increasing awareness of the value of pain research among clinicians, institutions, and policymakers; investing in sustainable research funding and infrastructure; strengthening training in research methods, statistics, and scientific writing; providing language and editorial support; and prioritising the development/translation and validation of patient-reported outcome measures in local languages. Collectively, such approaches are essential to improving the quality, credibility, and equity in pain research globally.

### 6.2. Educating communities about research

Education about research across multiple levels, from policymakers to researchers, healthcare professionals, and the public, is needed to improve the research ecosystems in LMICs. To address growing concerns about the trustworthiness of pain research, global efforts are needed to strengthen research capacity and support the use of ethical, rigorous, and cutting-edge methods. ENTRUST-PE (https://m.entrust-pe.org) is one international initiative working toward this goal.^69,71^ More targeted strategies are also required to support researchers in LMICs who lack local infrastructure, mentorship, and institutional resources.

### 6.3. Collecting equity-relevant sociodemographic data

Equity-relevant sociodemographic data are often missing in pain research, limiting our ability to identify predictors of chronic pain, understand moderators of treatment effects, and determine who study findings truly apply to. To address this gap, an international multistage eDelphi involving experts in health equity, pain researchers, healthcare professionals, and people with lived experience (n = 304; 45 countries) developed an 8-item minimum and 30-item optional dataset for use in all human pain research.^49,51^ Implementing these datasets can standardize data collection across studies and settings, enable cross-country comparisons, and support pooled analyses to better understand chronic pain and treatment responses.^52,53^

### 6.4. Equitable efforts to include diverse research participants

Traditional “equal” access approaches often exclude people who face linguistic, geographic, socioeconomic, or technological barriers from participation in (pain) research. About one-quarter of people in Australia and the United States do not speak English at home, yet English language–based eligibility criteria frequently prevent their participation in clinical pain research. As a result, groups most affected by chronic pain are frequently excluded from the very studies intended to inform their care.

Equitable research requires targeted strategies to include populations historically underrepresented in pain studies, such as people who speak nondominant language, those with limited access to recruitment sites, older adults, and individuals with multimorbidity. Harnessing artificial intelligence to develop multilanguage research materials can help recruit participants who are non-native speakers of the dominant languages. Achieving equity also demands shared responsibility across all interest holders—including researchers, healthcare professionals, institutions, journals, and funders—who must work collectively to reduce long-standing imbalances in research participation and representation.^6^

### 6.5. Avoiding colonizing pain research in low- and middle-income countries

Unfair and inequitable partnerships remain deeply embedded in global health research, often described as “colonizing global health.” Numerous analyses show stark under-representation of LMIC authors in research conducted in their own countries.^31,45,81^ For example, among 164 funded grants involving LMIC partners, more than half of the resulting publications (1242 of 2428; 51%) did not include any LMIC-affiliated authors.^31^ Similarly, a bibliometric review of studies conducted in sub-Saharan Africa found that 15% (4754 of 32,061) exhibited “parasite authorship”—research performed in LMICs with no LMIC authors at all.^81^ Even when LMIC researchers are included, they are under-represented in first and last authorship positions, with the lowest representation among authors from low-income countries.^29,81^ In response, some countries, such as China and India, have implemented strict data-sharing regulations that limit the movement of locally generated data beyond national borders. Although the reasons for these extreme measures are understandable, they may inadvertently hinder genuine collaboration and scientific progress.

To avoid repeating problems observed in the field of global health, the pain field must prioritize equitable, trust-building research partnerships in which all contributors—particularly LMIC investigators, who are often undervalued—are recognized, empowered, and appropriately credited.^1,66^ As collaborations between high- and lower-income countries continue to grow in pain field, we have a timely opportunity to adopt early safeguards and ethical governance mechanisms that prevent “colonizing” pain research.^1^ These safeguards will enhance the quality and relevance of pain research and care, ultimately supporting the development of a truly global and equitable research ecosystem.

### 6.6. Proposal for fair and impactful global health approach to pain research

Global partnerships are needed to advance impactful pain research by enabling the ethical sharing of scarce but important resources, including funding, expertise, capacity-building opportunities, and data. Such collaborations have been effective in other fields,^79^ offering models that the pain field can adopt, while also avoiding known mistakes, such as inequitable power-sharing, often reflected in authorship patterns.^1,29,45^ When grants originate in high-income countries, power is often retained. Research questions, methods, and authorship gravitate to institutions in the country of funder. This sidelines local priorities, working styles, and leaderships in LMICs^1,10,92^ while also compromising interpretation of the research findings.^8,55^

Effective partnership models are best cocreated with partners from low- and middle-income settings. The first priority is to operationalize power-sharing in practice, not merely in principle. This includes establishing joint principal investigators from both high- and lower-income settings, supported by agreements that leverage the complementary strengths of each partner while explicitly supporting research capacity in LMICs, as well as Aboriginal and migrant communities. Budgets should allocate dedicated resources for LMIC leadership, data analysis, and dissemination. Authorship plans should ensure that LMIC investigators hold leading roles commensurate with their contributions. Structured opportunities such as joint PhD scholarships or postdoctoral fellowships for LMICs partners can further strengthen capacity and sustain meaningful involvement.

Second, research must be led by or codesigned with local LMIC partners, or the target population (eg, Aboriginal and migrant communities in high-income countries) to ensure alignment with local contexts rather than direct transfer from high-income contexts. This requires using culturally adapted study materials; reducing participation burdens by partnering with communities to guide recruitment and study design (engaging groups historically excluded from research); adopting culturally appropriate outcome measures; testing locally codesigned or adapted interventions which is currently uncommon;^35^ and applying implementation-science approaches that reflect local workforce, financing, and delivery constraints. To develop successful and sustainable pain research programs in LMICs, the program needs to be led by LMIC-partners, and multiple examples are now available from many countries (eg, Argentina,^26^ Brazil,^5^ Ethiopia,^23^ Ghana,^4^ Pakistan,^54^ Nepal,^97^ Nigeria,^43^ South Africa^63,75^). Similarly genuine codesign of pain interventions has also started to emerge with Aboriginal peoples in high-income countries.^28,70^

Third, projects should build and sustain research capacity within LMICs by treating training, data systems, and sustainable roles as core components and explicit outcomes of the study, not afterthoughts. It is critical to build pain research on existing local knowledge, strengths, and capacity. Several successful research programs in multiple LMICs listed above have been through collaborations that included PhD scholarships of LMIC-partner within high-income countries and that aimed to advance pain knowledge in their home countries. True global health partnerships within pain research must address local priorities. Finally, peer-reviewed publications should report governance structures, budget allocations, authorship distribution, and community compensation—such transparency is a key component of reflexivity and shapes how readers interpret the conduct, power dynamic, and trustworthiness of the data and findings, which pain journals may reinforce (Table 2).

**Table 2:** Proposed best practices for global collaboration in pain research.

1. Stop helicopter and parasitic research practices: foster genuine collaboration, shared power, leadership, decision making, and equitable partnerships. Establish principal investigators (PI) across the countries (ie, high-income country PI and LMIC PI). Establish cogovernance structures with joint PIs across countries
2. Collaborate, contribute, mentor for the right reasons: the purpose and goals behind the collaboration should be codeveloped between high-income and low/middle-income country collaborators. These often should include developing (pain) research program and facilitate high research quality and capacity, particularly in the LMICs. But not at the cost of taking away opportunity from local LMIC partners to learn, capacity build, lead the work, and write the paper
3. Encourage, support, and empower the LMIC partners: in instances where LMIC partnership lack research experience, the high-income country partners should encourage LMIC partners to take the first and last authorship role. Offer PhD scholarships to LMIC partners can help ensure dedicated time for research, research training, and capacity building
4. Promote equitable leadership opportunities: offer opportunities especially for clinician-researchers, early to mid-career researchers, with the goal of research capacity building
5. Encourage LMIC-led funding proposals: LMIC-led funding applications can ensure capacity building in grant writing and ensure research budget allocated for local capacity building (eg, PhD scholarships and research assistants). High-income country partners should support LMIC-partners with opportunities for grant writing and leadership roles. LMIC partners should express their leadership interests and capacity
6. Harness the expertise and lived experience of LMIC diaspora in high-income countries: LMIC diaspora living and working in high-income countries are often disregarded as a valuable LMIC research contributor. Harnessing their unique understanding of local LMIC research/political/cultural/clinical context as well as best research practices in high-income countries can help bridge the gaps between countries and settings
7. Appropriate recognition of best practices in global pain research: academic promotions committees in high-income countries should reward researchers who promote capacity building in LMICs or Aboriginal or minority populations in high-income countries. For example, when high-income country partners are co-PIs on global pain research, their authorship roles as second or second last author should be recognized when there is a clear description of their supervisory/mentor roles in the manuscript. High-income country partners need to advocate this with their institutions and funders
8. Journal editors and reviewers must ensure fair authorship practices: editors and reviewers should question the authors when LMIC partners are not included as authors or are assigned middle-author roles for research that is conducted in LMICs. The same principle holds true for research conducted in Aboriginal and migrant populations in high-income countries. This point aligns with CRediT and ICMJE criteria for authorship

The first 7 points are relevant to high-income country partners, whereas the last point is directly relevant to the journal editors and reviewers.

CRediT, Contributor Roles Taxonomy; ICMJE, International Committee of Medical Journal Editors; LMICs, low- and middle-income countries.

Meaningful change requires a coordinated integration of top-down and ground-up approaches. An important top-down approach include reinforcement from the pain journals and other research outlets that mandate transparent reporting of authors' contributions from both high-income country and LMICs. Journals must investigate further to understand the reasons when local collaborators are excluded from authorship. Contributors from LMIC diaspora with high-income country affiliations should declare their worldviews through lived experiences in their reflexivity statements. Unless global health research partnerships prioritize transparency and accountability, they will fail to meaningfully support LMIC partners or achieve their intended goals. Table 3 includes a checklist for global pain research collaborators to use when planning, conducting, and reporting global health collaborative research.

**Table 3:** Checklist to incorporate principles for best practice in pain research in low- and middle-income settings.

	Key principles
□	Ensure the research question is cocreated with interest holders from the target populations*
□	Ensure the research project and methods are codesigned with interest holders from the target population*
□	Ensure best research methods suitable for the research question (including adequate sample sizes) while respecting/integrating local research approaches and practices
□	Consider broader selection criteria to include vulnerable and/or marginalized populations (minority language-speaking groups, Aboriginal and Indigenous groups, uneducated peoples, older adults, and/or those with low-socioeconomic positions)
□	Select outcome measures that are reliable, valid, and responsive in the target language and study populations
□	Ensure target population leadership and views throughout the research lifecycle from the conception through dissemination
□	Using strength-based approaches where applicable (by using key strengths of local interest holders and target populations)
□	Ensure the development core values and principles for research collaboration so that all involved agree to these values and principles before commencing research
□	Identify self-biases and actively avoid them influencing research questions, hypotheses, conduct, interpretation, writing, and dissemination
□	Plan or tailor the research to suit the local contexts where the research is being conducted (eg, recruitment and data collection approaches)
□	Ensure capacity building/strengthening among the target populations*
□	Consider strategies for sustainability and scale up early
□	Include reflexivity statements to ensure worldviews and positionality of all those who are involved at all steps of the projects (including LMIC diaspora)
□	Appropriately acknowledge the contributor to the research projects (eg, research assistants who collected data). Offer an opportunity for them to coauthor the resultant publication(s) when applicable
□	Ensure transparent, open, trustworthy research and its reporting (see https://m.entrust-pe.org). Practice open science principles
□	Use appropriate reporting guidelines to report the paper (see https://www.equator-network.org). Facilitate replication and reproducibility

Low- and middle-income settings include low- and middle-income countries and minority and aboriginal populations in high-income countries.

* Interest holders from the target populations include people with lived pain experience, multidisciplinary healthcare professionals, and researcher with experience in local research.

## 7. Conclusions

Ultimately, equitable pain research and clinical care share a single core mission to ensure the benefits of scientific discovery and evidence-based treatments extend to all individuals and communities affected by pain, regardless of geography, language, or cultural and spiritual beliefs. Achiving equitable pain research and care globally requires coordinated action across the entire research and health ecosystem from—public, patients, healthcare professionals, and researchers in high-income countries and LMICs to funders, pain organisations, and journal editors and reviewers.

These collective efforts must address the full range of drivers of inequity, including social, political, environmental, and commercial determinants. Applying a broader equity lens is essential to understand and reduce pain inequities and disparities in pain outcomes.^50^ Recent publications offer further recommendations.^96,99^ Strengthening health systems is also critical to addressing the growing global burden of pain, with detailed roadmaps available elsewhere.^9,14–16,88^ In parallel, strengthening research capacity in LMICs through codesigned approaches that harness local strengths is essential for generating contextually relevant evidence and addressing unmet needs and requires sustained global support and partnerships.^92^

Effective global pain research partnerships must foster shared leadership, empower LMIC researchers, and support cocreation across all research stages—from priority setting and study design to implementation and dissemination. Ultimately, knowledge must be generated from low- and middle-income settings to inform pain care globally. Such collaborative models move the field beyond regional boundaries, advancing global scientific efforts in which all partners contribute, learn, and benefit.

## Conflict of interest statement

The author has no conflicts of interest to declare (please also refer to the reflexivity statement within the paper).
